# Findings of virtual bronchoscopic navigation can predict the diagnostic rate of primary lung cancer by bronchoscopy in patients with peripheral lung lesions

**DOI:** 10.1186/s12890-022-02071-2

**Published:** 2022-07-14

**Authors:** Atsushi Kitamura, Yutaka Tomishima, Ryosuke Imai, Naoki Nishimura, Kohei Okafuji, Shosei Ro, Torahiko Jinta, Tomohide Tamura

**Affiliations:** Department of Respiratory Medicine, Thoracic Center, St. Luke’s International Hospital, Akashicho 9-1, Chuo City, Tokyo, 104-8560 Japan

**Keywords:** Bronchoscopy, Diagnostic rates, Lung cancer, Peripheral lung lesions, Virtual bronchoscopic navigation

## Abstract

**Background:**

Despite being minimally invasive, bronchoscopy does not always result in pathological specimens being obtained. Therefore, we investigated whether virtual bronchoscopic navigation (VBN) findings were associated with the rate of diagnosis of primary lung cancer by bronchoscopy in patients with peripheral lung lesions.

**Methods:**

This study included patients with suspected malignant peripheral lung lesions who underwent bronchoscopy at St. Luke’s International Hospital between October 2013 and March 2020. Patients diagnosed with primary lung cancer were grouped according to whether their pathology could be diagnosed by bronchoscopy, and their clinical factors were compared. In addition, the distance between the edge of the lesion and the nearest branch (“distance by VBN”) was calculated. The distance by VBN and various clinical factors were compared with the diagnostic rates of primary lung cancer.

**Results:**

The study included 523 patients with 578 lesions. After excluding 55 patients who underwent multiple bronchoscopies, 381 patients were diagnosed with primary lung cancer. The diagnostic rate by bronchoscopy was 71.1% (271/381). Multivariate analysis revealed that the lesion diameter (odds ratio [OR] 1.107), distance by VBN (OR 0.94) and lesion structure (solid lesion or ground-glass nodule; OR 2.988) influenced the risk of a lung cancer diagnosis. The area under the receiver operating characteristic curve for diagnosis based on lesion diameter and distance by VBN was 0.810.

**Conclusion:**

The distance by VBN and lesion diameter were predictive of the diagnostic rates of primary lung cancer by bronchoscopy in patients with peripheral lung lesions.

## Background

In recent years it has become common to use virtual bronchoscopic navigation (VBN) and endobronchial ultrasonography using a guide sheath (EBUS-GS) in the diagnosis of peripheral lung lesions by bronchoscopy. However, bronchoscopy is a minimally invasive procedure, and it is not always possible to obtain pathological specimens. The sensitivity of bronchoscopy for primary lung cancer depends on lesion size, with reports that sensitivity can be as low as 34% in patients with lesions < 2 cm [1]. In addition, computed tomography (CT) is usually performed before bronchoscopy to identify the bronchus involved in the lesion and to assess the presence of the “CT bronchus sign”. The presence of the CT bronchus sign is used to determine whether the lesion can be reached by bronchoscopy and to clarify the indication for bronchoscopy [2]. However, this method is subject to interobserver variability [3].

Conversely, if the diagnosis can be predicted more objectively before bronchoscopy based on data obtained by VBN and other methods, decisions regarding indications for bronchoscopy will also be more objective. If the diagnostic rate is expected to be low, procedures other than bronchoscopy, such as CT-guided needle biopsy or surgical lung biopsy, can be recommended.

SYNAPSE VINCENT^®^ version 5.5 (Fujifilm Medical Systems, Tokyo, Japan) has various applications, including surgical assistance, and one of its functions is virtual bronchoscopy. When the target lesion is marked, the software automatically identifies the nearest bronchus and calculates the shortest path to the lesion. The distance between the edge of the lesion and the nearest bronchus (“distance by VBN”) is also automatically calculated [4, 5]. The aim of the present study was to investigate whether the distance by VBN and clinical factors obtained before bronchoscopy are associated with the rate of primary lung cancer diagnosis by bronchoscopy.

## Methods

### Study subjects

This was a retrospective cohort study conducted at a single centre. The study included patients with suspected malignant peripheral lung lesions who underwent bronchoscopy at St. Luke’s International Hospital between October 2013 and March 2020.

Patients who had undergone multiple bronchoscopies were excluded from the study, as were patients who received a diagnosis other than primary lung cancer.

### Bronchoscopy and sedation

At St. Luke’s International Hospital, CT was performed at a thickness of 1.0 or 1.25 mm, and a VBN image was created on the basis of the CT data using LungPoint (Broncus Medical, Mountain View, CA, USA) [6]. All bronchoscopy procedures were performed using VBN by LungPoint and EBUS-GS. Radial EBUS was performed in all patients using an endoscopic ultrasound system (EU-ME1; Olympus, Tokyo, Japan) equipped with 20-MHz mechanical radial-type probes with a diameter of 1.4 mm (UM-S20-17S; Olympus) or 1.7 mm (UMS20-20R; Olympus). A thin bronchoscope (channel diameter, 2.0 mm; BF-P290 or BF-P260; Olympus) and GS (external diameter, 1.95 mm; K-201; Olympus) were used for the 1.4-mm probe, whereas a larger bronchoscope (channel diameter, 2.9 mm; BF-1T290 or BF-1T260; Olympus) and GS (external diameter, 2.55 mm; K-203; Olympus) were used for the 1.7-mm probe. The appropriate probes and bronchoscopes were selected by the operator (a respiratory specialist).

After brushing cytology and transbronchial aspiration cytology (TBAC) of the peripheral lesion, one cytological specimen was evaluated by rapid onsite cytology. After collection of the cytology specimen, biopsies were taken with forceps under fluoroscopic guidance for histopathological examination. Biopsies were repeated until specimens of adequate number and size were collected. The procedures followed those of Kitamura et al. [7].

All procedures were performed under local anaesthesia and sedation with intravenous midazolam and pethidine.

### Study design

At a different time to the actual bronchoscopy procedures, we compared associations of the distance by VBN, determined using SYNAPSE VINCENT, with the diagnosis rate of primary lung cancer and clinical factors. In addition, one observer (A.K.; respiratory specialist) reviewed the CT images and assessed the presence of the CT bronchus sign based on the location of the nearest branch and lesion [2]. Clinical factors included age, sex, lesion diameter, lesion structure, presence of the CT bronchus sign, EBUS-GS image (within, adjacent to, invisible) and pathological diagnosis. Lesion structure was classified as ground-glass nodule (GGN) or solid. GGN lesions included part-solid GGN and pure GGN lesions, whereas solid lesions included cavities, consolidation and nodules.

This study was approved by the Ethics Committee of St. Luke’s International Hospital (18-R177) on 27 February 2019.

### Follow-up and statistical analysis

For peripheral lesions that could not be diagnosed by bronchoscopy, pathological specimens were obtained (to the extent possible) using other methods, such as surgery or percutaneous needle biopsy. If a peripheral lesion could not be diagnosed by bronchoscopy and a mediastinal lymph node was accessible, we tried to establish a pathological diagnosis by EBUS-guided transbronchial needle aspiration from the bronchial lumen or endoscopic ultrasound-fine needle aspiration to the mediastinal lymph node from the oesophageal lumen. Patients who had no pathology diagnosis and refused to undergo further biopsy were diagnosed after at least 2 years of follow-up by both a radiologist and respiratory specialist. When the diameter of the lesion increased or the solid compartment of the GGN increased on CT, primary lung cancer was diagnosed without pathology.

Univariate analyses were performed using the Chi-squared test and Mann–Whitney *U* test, whereas multivariate analyses were conducted using logistic regression analysis. Two-sided *P* < 0.05 was regarded as statistically significant. All statistical analyses were conducted using R version 3.6.2 (R Foundation for Statistical Computing, Vienna, Austria), which was also used to draw the prediction graph. The statistical model was established using 80% of all patients (randomly selected). The statistical model comprised the results of bronchoscopy as an outcome variable, and the distance by VBN, lesion size and lesion structure as explanatory variables. The prediction graph was created based on this statistical model. The area under the receiver operating characteristic curve (AUC) of the prediction graph was evaluated in the remaining 20% of patients.

## Results

**Fig. 1:**
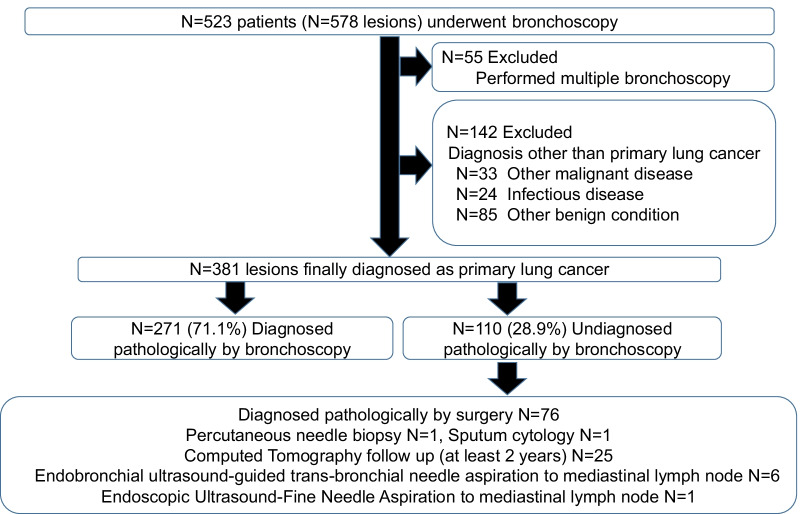
CONSORT flow diagram. Endobronchial ultrasound-guided transbronchial needle aspiration is a procedure used to puncture mediastinal lymph nodes through the bronchial lumen. Endoscopic ultrasound-fine needle aspiration is a procedure used to puncture mediastinal lymph nodes through the oesophageal lumen

In all, 523 patients (*n* = 578 lesions) underwent bronchoscopy. However, 55 patients who underwent multiple bronchoscopies were excluded from the study, as were a further 142 patients who received diagnoses other than primary lung cancer. Thus, 381 patients were finally diagnosed with primary lung cancer and were included in the study. The pathological diagnosis was made by bronchoscopy in 271 patients; thus, the diagnostic rate of primary lung cancer by bronchoscopy was 71.1%. Of the lesions that could not be diagnosed by bronchoscopy, 19.9% (76/381) were diagnosed by surgery and 8.9% (34/381) were diagnosed by other methods (Fig. 1). In 25 cases, patients refused further biopsy and the diagnosis of primary lung cancer was made without pathology. The solid component in the GGN increased slightly in 18 patients, whereas increased lesion size in nodules was found in seven patients.

**Fig. 2:**
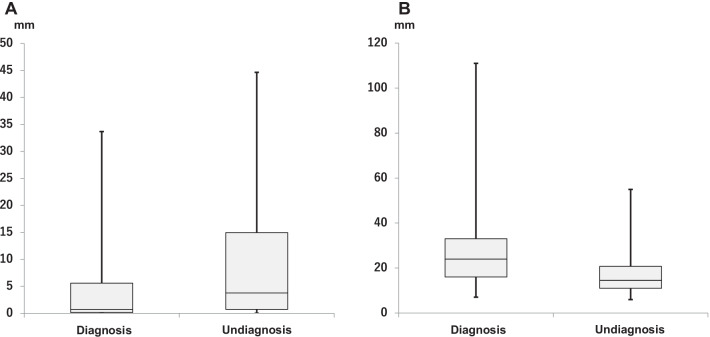
Box plots showing **A** the distance by virtual bronchoscopic navigation (VBN) and **B** lesion diameter in patients who were and were not diagnosed by bronchoscopy. Note, the distance by VBN refers to the distance between the edge of the lesion and the nearest bronchus, and is automatically calculated by SYNAPSE VINCENT. The boxes show the interquartile range, with the median value indicated by the horizontal line; whiskers show the range

Patient characteristics are presented in Table 1. Clinical factors were compared between patients with and without bronchoscopic diagnosis of malignancy. Multivariate analysis showed that lesion location, age and sex were not associated with an increased risk of a lung cancer diagnosis. However, lesion diameter, the distance by VBN and lesion characteristics were associated with the odds of a lung cancer diagnosis (Tables 1, 2). Specifically, the results suggest that the shorter the distance by VBN, the better the diagnostic rate for a lung cancer. The overall median distance by VBN was 1.17 mm. The median distance by VBN for the group diagnosed by bronchoscopy was 0.73 mm, compared with 3.78 mm for the group not diagnosed by bronchoscopy (Table 1; Fig. 2A). The overall median lesion diameter was 21 mm. The median lesion diameter in groups with and without a bronchoscopic diagnosis was 24 and 14.5 mm, respectively (Table 1; Fig. 2B). The diagnostic rate for cases that were positive for the bronchus sign was as high as 86.9%. Among patients with the bronchus sign, the median distance by VBN did not differ significantly between patients with and without a bronchoscopic diagnosis (0.53 vs 0.79 mm, respectively; Table 1). Conversely, among those without a bronchus sign, the median distance by VBN was significantly lower for patients with than without a bronchoscopic diagnosis (4.92 vs. 9.12 mm, respectively; *P* = 0.043; Table 1).

**Table 1:** Lesion characteristics

	All (*n* = 381)	Pathological diagnosis by bronchoscopy	
		Yes (*n* = 271)	No (*n* = 110)
Age (years)	71 [38–93]	71 [38–92]	71 [39–93]
Male sex	223	163 (60.1)	60 (54.5)
Lesion size (mm)	21 [6–111]	24 [7–111]	14.5 [6–55]
Lesion ≤ 20 mm	203	174 (64.2)	29 (26.4)
Lesion location			
Right upper lobe	126	88 (32.5)	38 (34.5)
S1		33 (12.2)	11 (10.0)
S2		32 (11.8)	15 (13.6)
S3		23 (8.5)	12 (10.9)
Right middle lobe	18	10 (3.7)	8 (7.3)
S4		5 (1.8)	6 (5.5)
S5		5 (1.8)	2 (1.8)
Right lower lobe	80	59 (21.8)	21 (19.1)
S6		25 (9.2)	11 (10.0)
S7		2 (0.7)	1 (0.9)
S8		14 (5.2)	2 (1.8)
S9		9 (3.3)	3 (2.7)
S10		9 (3.3)	4 (3.6)
Left upper lobe	104	76 (27.7)	28 (25.5)
S1 + 2		37 (13.7)	17 (15.5)
S3		21 (7.7)	10 (9.1)
S4		11 (4.1)	1 (0.9)
S5		7 (2.6)	0
Left lower lobe	53	38 (14.0)	15 (13.6)
S6		16 (5.9)	6 (5.5)
S8		7 (2.6)	2 (1.8)
S9		10 (3.7)	3 (2.7)
S10		5 (1.8)	4 (3.6)
Structure			
GGN	117	64 (23.6)	53 (48.2)
Solid	264	207 (76.4)	57 (51.8)
CT bronchus sign			
Present	245	213 (78.6)	32 (29.1)
Distance by VBN (mm)		0.53 [0–33.6]	0.79 [0.02–23.77]
Absent	136	58 (21.4)	78 (70.9)
Distance by VBN (mm)		4.92 [0.04–27.8]	9.12 [0.01–44.66]
EBUS-GS image			
Within	180	169 (62.4)	11 (10)
Adjacent to	82	62 (22.9)	20 (18.2)
Invisible	119	40 (14.8)	79 (71.8)
Diagnosis			
Adenocarcinoma	275	210 (77.5)	65 (59.1)
Squamous cell carcinoma	48	35 (12.9)	13 (11.8)
Small cell carcinoma	19	15 (5.5)	4 (3.6)
Atypical carcinoid	4	3 (1.1)	1 (0.9)
Adenosquamous carcinoma	3	3 (1.1)	0
Non-small cell carcinoma	2	1 (0.4)	1 (0.9)
Large cell carcinoma	1	0	1 (0.9)
Mucoepidermoid	1	1 (0.4)	0
Neuroendocrine tumour	2	2 (0.7)	2 (1.8)
Pleomorphic carcinoma	1	1 (0.4)	0
Unknown	25	0	25 (22.7)
Distance by VBN (mm)	1.17 [0–44.66]	0.73 [0–33.69]	3.78 [0.01–44.66]

Unless indicated otherwise, data are given as the median [range] or *n* (%). The distance by VBN refers to the distance between the edge of the lesion and the nearest bronchus, automatically calculated by virtual bronchoscopic navigation (VBN)

*EBUS-GS* endobronchial ultrasonography using a guide sheath, *GGN* ground-glass nodules

**Table 2:** Logistic regression analysis of factors affecting diagnosis by bronchoscopy

Variables	Univariate analysis	Multivariate analysis		
	OR (95% CI)	*P* value	OR (95% CI)	*P* value
Lesion location				
Right upper lobe	1.05 (0.47–2.27)	0.56	–	
Right middle lobe	0.7 (0.21–2.46)	0.68	–	
Right lower lobe	1.2 (0.50–2.85)	0.56	–	
Left upper lobe	1.29 (0.56–2.89)	0.91	–	
Left lower lobe	Reference			
Structure				
Solid	2.68 (1.60–4.50)	< 0.01	2.99 (1.67–5.43)	< 0.01
GGN	Reference			
Lesion size	1.11 (1.08–1.16)	< 0.01	1.11 (1.07–1.15)	< 0.01
Distance by VBN	0.93 (0.90–0.96)	< 0.01	0.94 (0.91–0.97)	< 0.01

The distance by VBN refers to the distance between the edge of the lesion and the nearest bronchus, automatically calculated by virtual bronchoscopic navigation (VBN)

*CI* confidence interval, *GGN* ground-glass nodules, *OR* odds ratio

**Fig. 3:**
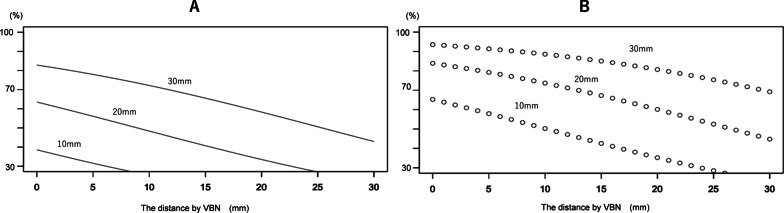
Graphs of the predicted diagnostic rate of primary lung cancer based on lesion diameter (10, 20 and 30 mm) and the distance by virtual bronchoscopic navigation (VBN) in patients with **A** ground-glass nodules and **B** solid lesions. Note, the distance by VBN refers to the distance between the edge of the lesion and the nearest bronchus, and is automatically calculated by SYNAPSE VINCENT

A graph was created to predict the diagnostic rate based on lesion diameter and distance by VBN. In patients with GGNs, the diagnostic rate of primary lung cancer by bronchoscopy was approximately 40% if the lesion diameter was 10 mm and the distance by VBN was 0 mm. In contrast, the diagnostic rate of primary lung cancer by bronchoscopy approached 80% when the lesion diameter was 30 mm and the distance by VBN was 0 mm (Fig. 3A). In patients with solid lesions, the diagnostic rate of primary lung cancer by bronchoscopy was 60% if the lesion diameter was 10 mm and the distance by VBN was 0 mm. Conversely, the diagnostic rate of primary lung cancer by bronchoscopy exceeded 90% when the lesion diameter was 30 mm and the distance by VBN was 0 mm (Fig. 3B). The AUC for diagnosis was 0.810.

There were no serious complications associated with bronchoscopy in the present study.

## Discussion

The distance by VBN and lesion diameter were significantly associated with a higher diagnostic rate of primary lung cancer (Table 2; Fig. 3). The AUC of the diagnostic prediction graph was 0.810, and the predictive accuracy appeared to be extremely high. The data from this study are extremely useful because they may enable data-based decisions to be made regarding bronchoscopic indications in patients. For example, surgery may be recommended after bronchoscopic diagnosis for lesions expected to have a high primary lung cancer diagnosis rate based on these diagnostic prediction graphs, whereas other procedures, such as CT guided biopsy, should be considered for lesions expected to have a low diagnostic rate.

GGN-type lesions are often less malignant and more difficult to identify than solid lesions, even under fluoroscopy, and so it is often difficult to make a pathological diagnosis using a small bronchoscopic specimen [8]. The present study showed that solid lesions were more predictive of a diagnosis of primary lung cancer in multivariate analysis than GGNs (Table 2). Therefore, the predictive graphs for diagnosis were created separately for GGNs and solid lesions.

Solid lesions that were 20 mm in diameter with a distance by VBN of 20 mm had a diagnostic yield of 60–70% (Fig. 3B). It is possible that there were branches that could not be read by VBN, and such lesions could be reached using innovative procedures such as curettage and TBAC. It remains important to use fluoroscopic techniques during an actual bronchoscopy.

LungPoint has been used at St. Luke’s International Hospital since 2013. To reduce the influence of VBN software, LungPoint was used instead of SYNAPSE VINCENT during the actual bronchoscopy. However, we have reported that SYNAPSE VINCENT has better reproducibility of peripheral branches than LungPoint [7]. In addition, LungPoint is no longer supported, will not be upgraded and cannot be used by new users. SYNAPSE VINCENT is continuously being upgraded and will continue to be used in our clinical practice. Therefore, in the present study, the diagnostic prediction was analysed using SYNAPSE VINCENT rather than LungPoint.

If all cases of suspected lung cancer before bronchoscopy were included in the present study, some cases would also include benign disease, making accurate evaluation more difficult. Thus, this study included a cohort of cases with either a final pathological diagnosis of primary lung cancer or without a pathological diagnosis but with a diagnosis of primary lung cancer after 2 years of CT follow-up.

The CT bronchus sign is a finding that predicts a high diagnostic yield before bronchoscopy [2]. In the present study, the diagnostic rate was 86.9% in patients with the CT bronchus sign (Table 1). However, judgement of the presence of the CT bronchus sign varies among readers, making it less objective [3]. In the present study, in patients positive for the bronchus sign, there was no significant difference in distance by VBN between those with and without bronchoscopic pathology. However, there was a significant difference in distance by VBN between patients with and without bronchoscopic pathology among those negative for the bronchus sign. The distance by VBN can be a more objective predictor of diagnosis in patients negative for the bronchus sign. The findings of this study suggest that the distance by VBN and lesion size, as more objective markers, can replace the CT bronchus sign for predicting the diagnosis.

## Conclusions

The distance by VBN and lesion diameter can predict the diagnostic rates of primary lung cancer by bronchoscopy in patients with peripheral lung lesions.

## Data Availability

The datasets used and analysed during the present study are available from the corresponding author upon reasonable request.

## Abbreviations

AUC: Area under the receiver operating characteristic curve
CT: Computed tomography
EBUS-GS: Endobronchial ultrasound-guide sheath
GGN: Ground-glass nodule
OR: Odds ratio
TBAC: Transbronchial aspiration cytology
VBN: Virtual bronchoscopic navigation
